# Gender differences in the association between self-reported stress and cigarette smoking in Korean adolescents

**DOI:** 10.1186/s12971-016-0084-9

**Published:** 2016-06-03

**Authors:** Kisok Kim, Hyejin Park

**Affiliations:** College of Pharmacy, Keimyung University, Daegu, 42601 Republic of Korea; Department of International Medical Management, Catholic University of Daegu, Kyungbuk, 38430 Republic of Korea

**Keywords:** Cigarette smoking, Perceived stress, Gender, Adolescent

## Abstract

**Background:**

The aim of this study was to examine the relationship between stress and smoking among Korean adolescents, as well as the influence of gender on this relationship.

**Methods:**

A cross-sectional study was conducted using data from 3930 adolescents aged 12–18 years, collected in the 2007–2012 Korea National Health and Nutrition Examination Surveys.

**Results:**

An increased level of self-reported stress was positively associated with increasing levels of smoking in both girls and boys (*p* for trend < 0.001). After adjusting for age, the odds ratios of smoking among girls and boys reporting very high levels of stress were 15.99 (95 % confidence interval (CI), 4.17–61.30) and 2.34 (95 % CI, 1.07–5.11), respectively, compared with those who reported low levels of stress.

**Conclusions:**

This study found a statistically significant association between stress and smoking among Korean adolescents and this association was stronger in girls than boys. Further research is needed to understand more fully the link between stress and smoking in adolescents, with particular attention to sex differences.

## Background

Smoking remains an important health concern and is responsible for 5.4 million deaths per year worldwide [1]. The prevalence of cigarette smoking is highest for young adults aged 18–25 years; however, smoking initiation tends to occur during adolescence [2]. Smoking prevalence in adolescent Korean girls decreased from 8.9 % in 2005 to 4.0 % in 2014. However, the smoking rate by adolescent boys has remained at 14.0–17.4 % over the past 10 years [3]. Therefore, it is important to identify factors that may affect the initiation and maintenance of smoking in adolescent populations.

Stress is one of the major factors that have been consistently correlated with adolescents’ smoking. Multiple studies have reported an association between smoking and mental health conditions in specific population groups [4]. For example, a recent study conducted in China reported that subjects with high perceived life stress and high perceived work stress show 45 and 75 % excess odds ratios for smoking, respectively, compared with that of a low-stress group [5]. Although stress plays an important role in cigarette smoking among some adolescent populations, it is unclear whether the association is true across different gender groups [6, 7].

Adolescents experience many stressful life events, including study concerns, financial problems and changes in identity and relationships with parents and peers [8]. In particular, Korean adolescents have extreme stress owing to both their academic and social demands. For example, 7 out of 10 Korean high-school students perceive that they are stressed because of academic demands and suicide is the leading cause of death among Korean adolescents [9]. However, relatively little information is available about the effect of stress on smoking in Korean adolescents, especially among each gender.

This study examined the influence of gender on the association between self-reported stress and cigarette smoking among Korean adolescents. Data were obtained from the 2007–2012 Korea National Health and Nutrition Examination Survey (KNHANES), a nationally representative survey conducted in the Republic of Korea.

## Methods

### Participants and procedures

This study was based on data from the fourth and fifth waves (2007–2012) of KNHANES, provided by the Korea Centers for Disease Control and Prevention. Samples from KNHANES were selected using a stratified, multi-stage, cluster-sampling design with proportional allocation based on the National Census Registry. Detailed information on the survey design and sampling procedures has been reported elsewhere [10]. Using weighted data from the 2007–2012 KNHANES databases, 3930 adolescents aged 12–18 years who had no missing responses on the questionnaire were included in this study.

### Measures

KNHANES included questions on demographic and socioeconomic characteristics, psychological health and smoking habits. Information regarding stress and cigarette smoking was obtained by a self-administered questionnaire. The level of perceived stress was measured using the following question: “How much stress do you usually feel?” There were four possible answers: “only a little”, “to some extent”, “rather much” and “very much”, which were labelled as low, medium, high and very high levels of perceived stress, respectively. The number of days cigarettes were smoked and the number of cigarettes smoked per day in the past month were also asked in the survey. Cigarette smoking was defined as smoking more than one cigarette in the past month. The study protocol was approved by the Korean Ministry of Health and Welfare and was conducted in accordance with the Ethical Principles for Medical Research Involving Human Subjects, as defined by the Helsinki Declaration. All study participants provided informed written consent.

### Data analysis

As appropriate, the frequency, mean and 95 % confidence intervals (CIs) were calculated for each gender to describe the smoking habits according to categories of perceived stress. Logistic regression models were used to estimate the odds ratio (OR) and 95 % CIs for cigarette smoking status among participants who reported medium to very high stress compared with the reference group (those who reported low stress). The presence of a linear trend was evaluated by defining a linear contrast in each of the linear and logistic regression models. All statistical analyses were conducted using SAS v9.3 (SAS Institute, Cary, NC, USA). Statistical analyses accounted for the survey design, and appropriate procedures in SAS such as surveyfreq and surveylogistic were used with weighted data.

## Results

This study included 3930 adolescents aged 12–18 years, and the demographic characteristics of participants are shown in Table 1. Age, average household income, place of residence did not differ significantly according to sex, whereas smoking and stress rates were significantly different between boys and girls. The basic characteristics and smoking habits of the study population, sorted by severity of self-reported stress, are presented in Table 2. Among girls in the study, mean age was 14.7 years, and the overall average number of days cigarettes were smoked and the average number of daily cigarettes smoked in the past month were 0.64 and 0.27, respectively. Among boys, mean age was 14.6 years, with the overall average number of days cigarettes were smoked and average number of daily cigarettes smoked in the past month being 2.08 and 0.97, respectively. As the severity of stress increased, girls and boys were more likely to smoke more days and with more cigarettes per day (*p* for trend = 0.001 or < 0.001).

**Table 1:** Demographic characteristics of participants

Characteristics	Total (%)	Girls (%)	Boys (%)	*p*
Age (years)				0.808
12	697 (17.7)	317 (17.0)	380 (18.4)	
13	654 (16.6)	312 (16.7)	342 (16.6)	
14	658 (16.7)	313 (16.8)	345 (16.7)	
15	524 (13.3)	247 (13.2)	277 (13.4)	
16	507 (12.9)	251 (13.5)	256 (12.4)	
17	482 (12.3)	238 (12.8)	244 (11.8)	
18	408 (10.4)	188 (10.1)	220 (10.7)	
Average household income (US$/month)				0.748
< 1500	902 (23.0)	439 (23.5)	463 (22.4)	
1500–2499	1053 (26.8)	506 (27.1)	547 (26.5)	
2500–3500	771 (19.6)	359 (19.2)	412 (20.0)	
> 3500	1204 (30.6)	562 (30.1)	642 (31.1)	
Place of residence				0.953
Urban	3263 (83.0)	1550 (83.1)	1713 (83.0)	
Rural	667 (17.0)	316 (16.9)	351 (17.0)	
Cigarette smoking				<0.001
Yes	308 (7.8)	73 (3.9)	235 (11.4)	
No	3622 (92.2)	1793 (96.1)	1829 (88.6)	
Stress				<0.001
Very high	158 (4.0)	89 (4.8)	69 (3.3)	
High	911 (23.2)	480 (25.7)	431 (20.9)	
Medium	2250 (57.3)	1043 (55.9)	1207 (58.5)	
Low	611 (15.6)	254 (13.6)	357 (17.3)

**Table 2:** Demographic characteristics by categories of self-reported stress

	Total	Stress				*p* for trend
		Low	Medium	High	Very high	
Whole sample						
Number of day cigarette smoked per month, d (95 % CI)	1.40 (1.22–1.58)	0.77 (0.44–1.10)	1.19 (0.97–1.41)	1.94 (1.49–2.39)	3.68 (2.22–5.13)	<0.001
Number of cigarette smoked per day, cigs (95 % CI)	0.63 (0.52–0.75)	0.40 (0.10–0.71)	0.51 (0.38–0.64)	0.85 (0.59–1.10)	2.08 (1.07–3.10)	<0.001
Girls						
Number of day cigarette smoked per month, d (95 % CI)	0.64 (0.46–0.82)	0.35 (0.01–0.70)	0.42 (0.23–0.61)	0.91 (0.48–1.34)	2.72 (1.07–4.37)	<0.001
Number of cigarette smoked per day, cigs (95 % CI)	0.27 (0.18–0.36)	0.09 (0.01–0.18)	0.15 (0.08–0.21)	0.36 (0.17–0.55)	1.69 (0.39–2.98)	<0.001
Boys						
Number of day cigarette smoked per month, d (95 % CI)	2.08 (1.78–2.38)	1.07 (0.56–1.58)	1.85 (1.48–2.22)	3.08 (2.28–3.88)	4.91 (2.34–7.49)	<0.001
Number of cigarette smoked per day, cigs (95 % CI)	0.97 (0.77–1.16)	0.62 (0.11–1.14)	0.82 (0.59–1.05)	1.39 (0.90–1.88)	2.59 (0.95–4.24)	0.001

*CI* confidence interval

Table 3 shows the population-weighted prevalence and ORs of cigarette smoking by self-reported stress. A significant positive trend was observed between the level of stress and cigarette smoking prevalence (*p* for trend < 0.001), with 6.27 % of the adolescents that smoked belonging to the group that felt low levels of stress compared with 21.23 % in the group that felt very high levels of stress. Additionally, the adjusted ORs for cigarette smoking were positively correlated with increased self-reported stress (*p* for trend = 0.001). In girls, the trends in prevalence and adjusted ORs with increasing levels of stress were also positive for cigarette smoking (*p* for trend < 0.001). After adjusting for age, income and place of residence, the ORs for cigarette smoking were 2.67 (95 % CI, 0.76–9.35) among those who reported medium levels of stress, 4.13 (95 % CI, 1.21–14.15) among those reported high levels of stress, and 15.99 (95 % CI, 4.17–61.30) among those reporting very high levels of stress, compared with those who reported low level of stress. In boys, the trends in prevalence of cigarette smoking according to stress level were significant (*p* for trend = 0.013), while the trends of adjusted ORs were marginally significant (*p* for trend = 0.035). In boys, the adjusted ORs for cigarette smoking among the same stress groups were 1.60 (95 % CI, 0.95–2.69), 2.27 (95 % CI, 1.26–4.10) and 2.34 (95 % CI, 1.07–5.11), respectively, compared to the reference group.

**Table 3:** Weighted prevalence and adjusted odds ratios (95 % CI) of smoking by self-reported stress

Stress	Low	Medium	High	Very high	*p* for trend
Whole sample					
Prevalence, %	6.27	9.61	12.57	21.23	<0.001
AOR (95 % CI)	1.00 (reference)	1.49 (0.93–2.38)	1.93 (1.17–3.17)	3.56 (1.84–6.89)	0.001
Girls					
Prevalence, %	1.42	4.19	6.73	22.06	<0.001
AOR (95 % CI)	1.00 (reference)	2.67 (0.76–9.35)	4.13 (1.21–14.15)	15.99 (4.17–61.30)	<0.001
Boys					
Prevalence, %	9.57	14.13	18.87	19.96	0.013
AOR (95 % CI)	1.00 (reference)	1.60 (0.95–2.69)	2.27 (1.26–4.10)	2.34 (1.07–5.11)	0.035

*AOR* odds ratio adjusted for age, income and place of residence, *CI* confidence interval

## Discussion

In this nationally representative study, we found that the frequency and quantity of cigarette smoking increased with increased stress in Korean girls and boys. Previous studies also found a relationship between stress and various stages of smoking (including initiation) for multiracial/ethnic US adolescents [11–14] and Australian adolescents [15, 16], suggests that stress may be related to smoking for adolescents across different race/ethnic groups, including Korean adolescents.

We found that although the prevalence and ORs of cigarette smoking increased significantly with stress levels in girls and boys, smoking was more strongly associated with stress in girls. The prevalence of smoking among girls reporting “very high” stress was higher than that of boys also reporting “very high” stress. Although the biological mechanisms underlying these gender differences remain to be elucidated, one hypothesis is that women are more sensitive than men to the effects of cigarette smoking on the cortisol response to stress [6]. Future studies investigating the mechanisms underlying these gender differences are required to confirm and extend the results of this study.

The wide CIs as a result of the small sample size of smoking girls warrant caution when interpreting the findings. The prevalence of smoking by adolescent girls was only 3.9 %, which is much lower than that of boys. The wide CIs suggest that an additional study with a larger sample is likely to improve the effect-size estimates and clarify the effect of stress on smoking in girls.

The present study has several limitations. We relied on self-reports to assess stress levels, which may lead to misclassification and measurement errors [17]. Although the definition and measurement of stress remain the subject of debate, self-reporting of perceived stress provides a more appropriate measure of the actual levels of stress experienced by individuals than external counting of potential stressors [18]. Furthermore, the question we used to assess self-reported stress level is standard, and similar questions have been used in other studies, such as in Canadian and Korean nationwide health surveys, where self-perceived stress level was assessed by asking one question consisting of five response categories ranging from none to very high [19, 20]. Additionally, information about smoking habits was obtained via self-reports rather than direct observations, which may have led to reporting bias.

Despite these limitations, this is the first study assessing the association between stress and smoking in Korean adolescents using nationally representative data. Thus, with the advantages of systematic sampling, the results of this study can be generalized to all Korean adolescents. Another strength is that the present study provides not only the prevalence of smoking, but also quantitative information with regard to the smoking habits among Korean adolescents. Further research is needed to understand more fully the link between stress and smoking in adolescents, with particular attention to sex differences. Moreover, future studies will be important to assess the effectiveness of stratifying smoking intervention strategies for adolescents by stress levels.

## Conclusion

This study found that self-reported stress was strongly association with cigarette smoking among Korean adolescents and this association was stronger in girls than boys. Given that stress and smoking interfere with several physiological and pathological processes, our results suggest that both psychological and behavioral factors should be taken into account when developing interventions for improving health status of adolescents. In addition, an assessment of psychological factors among adolescent smokers, especially in girls, would be important to identify causal factors and to intervene earlier.

## References

[1] World Health Organization. Tobacco Free Initiative. 2015. http://www.who.int/tobacco/framework/cop/facts_and_figures_about_tobacco.pdf. Accessed 16 Aug 2015.

[2] U.S. Department of Health and Human Services. Preventing Tobacco Use Among Youth and Young Adults: A Report of the Surgeon General. 2012. http://www.surgeongeneral.gov/library/reports/preventing-youth-tobacco-use/full-report.pdf. Accessed 16 Aug 2015.

[3] Korea Center for Disease Control and Prevention. The Tenth Korea Youth Risk Behavior Web-Based Survey 2014. 2015. http://yhs.cdc.go.kr/new/?c=pds&s=1&gbn=viewok&sp=&sw=&ps=10&gp=1&ix=8. Accessed 16 Jan 2016.

[4] Fields S, Leraas K, Collins C, Reynolds B (2009). Delay discounting as a mediator of the relationship between perceived stress and cigarette smoking status in adolescents. Behav Pharmacol.

[5] Cui X, Rockett IR, Yang T, Cao R (2012). Work stress, life stress, and smoking among rural-urban migrant workers in China. BMC Public Health.

[6] Back SE, Waldrop AE, Saladin ME, Yeatts SD, Simpson A, McRae AL (2008). Effects of gender and cigarette smoking on reactivity to psychological and pharmacological stress provocation. Psychoneuroendocrinology.

[7] Mitic WR, McGuire DP, Neumann B (1985). Perceived stress and adolescents cigarette use. Psychol Rep.

[8] Dugan S, Lloyd B, Lucas K (1999). Stress and coping as determinants of adolescent smoking behavior. J Applied Soc Psychol.

[9] Statistics Korea. Youth statistics. 2014. http://kostat.go.kr/portal/eng/pressReleases/13/3/index.board. Accessed 16 Aug 2015.

[10] Yun BH, Choi YR, Choi YS, Cho S, Lee BS, Seo SK (2015). Age at first delivery and osteoporosis risk in Korean postmenopausal women: The 2008–2011 Korea National Health and Nutrition Examination Survey (KNHANES). PLoS One.

[11] Biafora FA, Vega WA, Warheit GJ, Gil AG (1994). Stressful life events and changes in substance use among multiracial/ethnic sample of adolescent boys. J Commun Psychol.

[12] Guthrie BJ, Young AM, Boyd CJ, Kintner EK (2001). Dealing with daily hassles: smoking and African-American adolescent girls. J Adolesc Health.

[13] Finkelstein DM, Kubzansky LD, Goodman E (2006). Social status, stress, and adolescent smoking. J Adolesc Health.

[14] Voorhees CC, Schreiber GB, Schumann BC, Biro F, Crawford PB (2002). Early predictors of daily smoking in young women: The national heart, lung, and blood institute growth and health study. Prevent Med.

[15] Byrne DG, Byrne AE, Reinhart MI (1995). Personality stress and the decision to commence cigarette smoking in adolescence. J Psychosom Res.

[16] Byrne DG, Mazanov J (2003). Adolescent stress and future smoking behaviour: A prospective investigation. J Psychosom Res.

[17] Day NE, Wong MY, Bingham S, Khaw KT, Luben R, Michels KB (2004). Correlated measurement error--implications for nutritional epidemiology. Int J Epidemiol.

[18] Nielsen NR, Zhang ZF, Kristensen TS, Netterstrøm B, Schnohr P, Grønbaek M (2005). Self reported stress and risk of breast cancer: prospective cohort study. BMJ.

[19] Kwon JA, Park EC, Lee M, Yoo KB, Park S (2013). Does stress increase the risk of atopic dermatitis in adolescents? results of the Korea Youth Risk Behavior Web-based Survey (KYRBWS-VI). PLoS One.

[20] Ramage-Morin PL, Shields M, Martel L (2010). Health-promoting factors and good health among Canadians in mid- to late life. Health Rep.

